# A meta‐analysis on racial disparity in administration of tissue plasminogen activator (tPA) in stroke patients

**DOI:** 10.1002/hsr2.2181

**Published:** 2024-07-01

**Authors:** Muhammad Omar Larik, Pratik Bhattarai

**Affiliations:** ^1^ Department of Medicine Dow International Medical College Karachi Pakistan; ^2^ Department of Medicine Manipal College of Medical Sciences Pokhara Nepal

**Keywords:** meta‐analysis, racial disparity, tissue plasminogen activator (tPA)

## 1 INTRODUCTION

Momentous advances in acute stroke therapy have been marked by the use of tissue plasminogen activators (tPA) within a 4.5‐h window period, which is now considered the standard method of treatment.^1^ Several studies have highlighted the potential racial disparities within the administration of tPA in patients of various races,^2^, ^3^, ^4^, ^5^ whereas other studies have not been able to detect such differences. It is exceedingly important to address all present racial disparities, to preserve equality and provide nondiscriminatory, quality care to all patients. In light of such controversial results, this meta‐analysis was performed to assess the differences within the administration of tPA between the Black and White race.

## 2 METHODS

PubMed and Scopus were extensively searched for potentially relevant studies, utilizing the following keywords: “racial disparity,” “racial differences,” “White,” “Black,” “tissue plasminogen activator,” “tPA,” “alteplase,” and “stroke.” The initial search yielded 412 results from inception to June 2023. All statistical analyses were performed using Review Manager (RevMan version 5.3; Copenhagen: The Nordic Cochrane Centre, The Cochrane Collaboration, 2014), using the random‐effects model and odd ratios to compare the data of the outcome of interest. Statistical significance was denoted at a*p*‐value < 0.05. Quality assessment was performed using the Newcastle–Ottawa scale for cohort studies, in which the studies were rated out of nine. Ethical approval was not required for this study, as data was extracted from published studies retrieved from online public databases.

## 3 RESULTS

**Figure 1:**
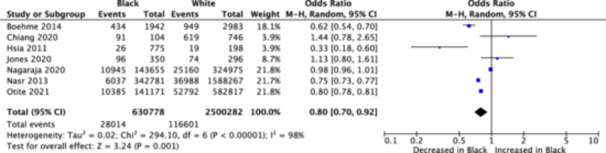
Forest plot of racial differences in administration of tPA. CI, confidence interval; tPA, tissue plasminogen activators.

After shortlisting, there were seven studies selected for inclusion within this meta‐analysis, featuring 3,131,060 participants (including 630,778 Black patients, and 2,500,282 White patients) receiving tPA after an ischemic stroke attack.^2^, ^3^, ^4^, ^5^, ^6^, ^7^, ^8^ This comprehensive meta‐analysis revealed that Black patients were significantly associated with a lower rate of administration of tPA in comparison to their White counterparts (odds ratio: 0.80; 95% confidence interval: 0.70–0.92; *p* = 0.001; *I* ^2^ = 98%; Figure 1). Overall, the quality of included studies was high and demonstrated a low risk of bias, as per the Newcastle–Ottawa scale for cohort studies. Baseline characteristics of the included study population are available in Table 1.

## 4 DISCUSSION

There has been an upward trend in the recognition of racial disparities within the field of modern medicine, such as the presence of racial disparities within clinical outcomes poststroke and postcardiac arrest.^9^, ^10^ With respect to the disparities observed within our meta‐analysis, this could be attributed to the lower rates of hospital arrival via ambulances among non‐White races, leading to a delayed treatment decision and subsequently a delay in anti‐stroke therapy, which puts the patients beyond the 4.5‐h timeframe for receiving tPA. Additionally, stroke awareness and symptom recognition plays a pivotal role in timely hospital arrival, which has been observed to be relatively weaker among the Black population.^11^ Contrastingly, such differences may be a result of increased comorbidities in the Black population, which is observed to have a 10% greater prevalence of hypertension compared to their White counterparts.^8^ The reasons for potential disparities have been summarized in a statement released by the American Heart Association.^11^

In light of the presence of disparities, it is imperative to embark on discussion and consolidate timely, effective interventions against the existing barriers in stroke care. Emphasis on various healthcare initiatives and policies is strongly recommended, such as the “Affordable Care Act” or the “Racial and Ethnic Approach to Community Health.” Moreover, the unquantified role of implicit bias within healthcare professionals has been a rising concern within the medical field, thus encouraging a greater racial diversity of the relevant staff and neurologists is always ideal.^10^

Although this brief analysis provides quantifiable information regarding the presence of racial disparities in tPA administration, further exploration is essential to establish at a robust conclusion. Firstly, the lack of data on other races, for example, Asian or Hispanic populations, limits the generalizability of these findings, and thus researchers are urged to formulate comparisons among other races, in addition to the popular Black versus White comparison. Secondly, publication of additional data in terms of age, gender, comorbidities, stroke subtype, and severity will permit future subgroup analyses, to precisely pinpoint target populations for necessary interventions. Ultimately, these aggregated findings set the stage for future trials and analyses that encompass a comprehensive sample pool, to accurately address such findings by implementing population and demographic‐specific interventional changes.

**Table 1:** Baseline characteristics of included study population.

Author (year)	Study design	Black, *n*	White, *n*	Median Age, y (IQR)		Female, *n* (%)		Hypertension, *n* (%)				DM, *n* (%)		AF, *n* (%)		NOS Rating
				Black	White	Black	White	Black		White		Black	White	Black	White	
Boehme (2014)	Retrospective	1942	2983	62 (44–83)	67 (35–78)	2372 (48.2)		1569 (81.6)			2089 (70.2)	686 (35.5)	888 (29.8)	197 (10.2)	573 (19.2)	********
Chiang (2020)	Retrospective	104	746	‐												********
Hsia (2011)	Retrospective	775	198	‐	‐	538 (55.3)			‐	‐		‐	‐	‐	‐	********
Jones (2020)	Prospective	350	296	44 (37–47)	42 (35–47)	154 (44.0)	125 (42.0)		181 (52.2)	95 (32.3)		75 (21.5)	37 (12.6)	11 (3.2)	9 (3.1)	********
Nagaraja (2020)	Retrospective	143,655	324,975	66 (56–76)	73 (63–83)	72,402 (50.4)	165,087 (50.8)		127,997 (89.1)	172,202 (84.1)		73,120 (50.9)	120,566 (37.1)	20,399 (14.2)	70,195 (21.6)	********
Nasr (2013)	Retrospective	342,781	1,588,267	70 (50–91)	62 (39–84)	191,812 (56.0)	868,226 (54.7)		4787 (79.3)	25,978 (70.2)		1880 (31.2)	8354 (22.5)	1060 (17.6)	12,355 (33.4)	********
Otite (2021)	Retrospective	141,171	582,817	‐	‐	468,009 (51.6)			738,494 (81.5)			324,563 (35.8)		219,211 (24.2)		*******

Abbreviations: AF, atrial fibrillation; DM, diabetes mellitus; IQR, interquartile range; *n*, number of participants; NOS, Newcastle–Ottawa Scale; y, year.

There are several limitations that must be highlighted. Firstly, the exclusive retrospective and observational nature of the included studies leads to residual bias and contributes to significant heterogeneity, potentially demeriting the findings. However, the retrospective inclusion results in immensely larger sample sizes. Secondly, the comparison between the Black versus White races highlights important disparities; however, true remarks regarding the racial disparities cannot be produced without considering other races, for example, patients of Asian or Hispanic descent. Thirdly, the global inclusion of data leads to marginal inconsistencies and heterogeneity within our results, potentially due to the regional variation in prevalence of comorbidities, or the differing modalities of treatment.

## 5 CONCLUSION

In conclusion, patients of the Black race were significantly associated with a lower administration of tPA in comparison to their White counterparts. It is recommended to encourage greater emphasis and development on various healthcare initiatives striving for racial inclusion. Moreover, the increase of racial diversity within the medical field, especially within neurology, is important to curb the risk of implicit bias against patients of the other races.

## AUTHOR CONTRIBUTIONS

**Muhammad Omar Larik**: Conceptualization; methodology; formal analysis; writing—original draft; writing—review & editing. **Pratik Bhattarai**: Formal analysis; writing—original draft; writing—review & editing.

## CONFLICT OF INTEREST STATEMENT

The authors declare no conflict of interest.

## TRANSPARENCY STATEMENT

The lead author Pratik Bhattarai affirms that this manuscript is an honest, accurate, and transparent account of the study being reported; that no important aspects of the study have been omitted; and that any discrepancies from the study as planned (and, if relevant, registered) have been explained.

## Data Availability

The data that support the findings of this study are available from the corresponding author upon reasonable request. The authors confirm that the data supporting the findings of this study are available within the article.
